# Co-expression of growth regulator genes *HvWUS* and *HvBBM2* enhances barley transformation efficiency

**DOI:** 10.1093/plphys/kiaf494

**Published:** 2025-10-28

**Authors:** Xinyi He, Danyin Huang, Jie Yao, Zhiye Gu, Shengguan Cai, Qiufang Shen, Guoping Zhang, Lingzhen Ye

**Affiliations:** Institute of Crop Science, Key Laboratory of Crop Germplasm Resource of Zhejiang Province, Zhejiang University, Hangzhou 310058, China; Institute of Crop Science, Key Laboratory of Crop Germplasm Resource of Zhejiang Province, Zhejiang University, Hangzhou 310058, China; Institute of Crop Science, Key Laboratory of Crop Germplasm Resource of Zhejiang Province, Zhejiang University, Hangzhou 310058, China; Institute of Crop Science, Key Laboratory of Crop Germplasm Resource of Zhejiang Province, Zhejiang University, Hangzhou 310058, China; Institute of Crop Science, Key Laboratory of Crop Germplasm Resource of Zhejiang Province, Zhejiang University, Hangzhou 310058, China; Zhongyuan Institute, Zhejiang University, Zhengzhou 450000, China; Institute of Crop Science, Key Laboratory of Crop Germplasm Resource of Zhejiang Province, Zhejiang University, Hangzhou 310058, China; Zhongyuan Institute, Zhejiang University, Zhengzhou 450000, China; Institute of Crop Science, Key Laboratory of Crop Germplasm Resource of Zhejiang Province, Zhejiang University, Hangzhou 310058, China; Zhongyuan Institute, Zhejiang University, Zhengzhou 450000, China; Institute of Crop Science, Key Laboratory of Crop Germplasm Resource of Zhejiang Province, Zhejiang University, Hangzhou 310058, China; Zhongyuan Institute, Zhejiang University, Zhengzhou 450000, China

## Abstract

Co-expression of HvWUS and HvBBM2 that regulate plant growth substantially boosts the efficiency of genetic transformation and genome editing across diverse barley varieties, facilitating crop research and improvement.

Dear Editor,

A stable and efficient genetic transformation system is essential for functional genome research and crop breeding. Immature embryo of a barley (*Hordeum vulgare* L.) variety Golden Promise (GP) is considered the most suitable explant, and the *Agrobacterium*-mediated transformation is the most commonly used for delivering T-DNA (Bartlett et al. 2008; Harwood 2014). However, apart from GP, only a few barley genotypes, such as Schooner, Chebec, Sloop, and Vlamingh have been successfully transformed, albeit with very low efficiency (Murray et al. 2004; Zang et al. 2022). One of the most important limiting factors is regeneration. In short, the efficiency of genetic transformation in barley remains quite low, presenting a significant bottleneck for functional genome research and breeding efforts.

Recently, the transformation efficiencies of many crops have been significantly improved through overexpressing the genes of plant growth regulators. For example, Lowe et al. (2016) enhanced maize (*Zea mays* L.) transformation efficiency by overexpressing *WUSCHEL2* (*WUS2*) and *BABY BOOM* (*BBM*). Similarly, Wang et al. (2022) achieved a significant improvement in wheat (*Triticum aestivum* L.) transformation by expressing another *WUSCHEL*-related homeobox gene, *WOX5*. Additionally, the overexpression of *GROWTH-REGULATING FACTOR 4* (*GRF4*) and its interacting partner *GIF1* has been shown to significantly increase transformation efficiencies in wheat (Debernardi et al. 2020). This raises the question: Can the efficiency of barley transformation be improved by expressing genes that encode plant growth regulators? To date, little relevant research has been conducted in barley.

In this study, we compared the effects of various plant growth regulators on barley transformation and successfully enhanced transformation efficiency by co-expressing *HvWUS* (*HORVU.MOREX.r3.2HG0201080*) and *HvBBM2* (*HORVU.MOREX.r3.3HG0305370*). At first, we identified homologous genes of *ZmWUS2*, *ZmBBM*, *TaGRF4*, and *TaGIF1* in barley (Supplementary Fig. S1 and Table S1), and found the differences in the structural domain sequences of these genes between barley and other species (Supplementary Fig. S2). Previously, another *WUS* and *BBM* genes were already reported by Suo et al. (2021), but phylogenetic analyses showed that they were not the closest homologous genes to *ZmWUS2* and *ZmBBM* (Supplementary Fig. S1). Taken together, we constructed 6 vectors containing *HvGRF4-HvGIF1*, *TaGRF4-TaGIF1*, *HvWUS* + *HvBBM2*, *ZmWUS2* + *ZmBBM*, *HvWOX5* + *HvBBM2*, respectively and a control vector (CK) (Fig. 1A) and used them to transform immature embryos of the GP genotype, following the transformation protocol described by Harwood (2014). Green fluorescent protein ZsGreen (Lowe et al. 2016) was used to identify the positive callus. The results showed that the positive rates (positive callus/callus) of *HvGRF4-HvGIF1*, *TaGRF4-TaGIF1*, *HvWUS* + *HvBBM2* and *ZmWUS2* + *ZmBBM* vectors were significantly higher than those of the control (Fig. 1B, Supplementary Fig. S3A).

**Figure 1.**
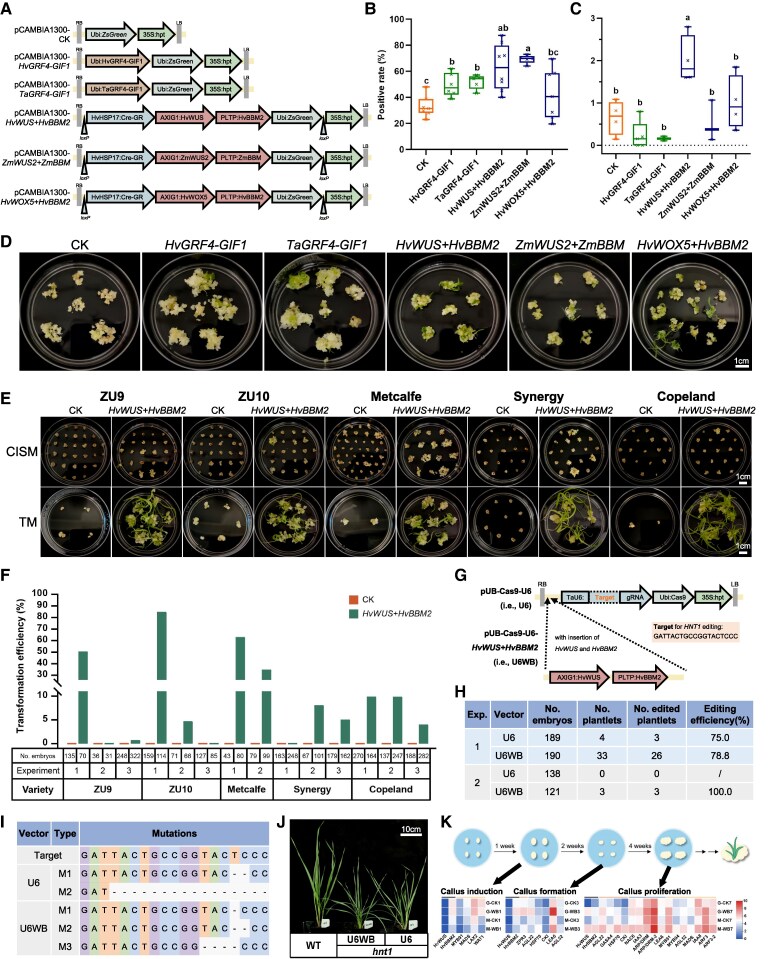
Co-expression of *HvWUS* and *HvBBM2* enhances regeneration of transformed barley plants. **A)** Schematic overview of the vectors utilized in this study. Positive rates (positive callus/callus) (*n* = 5 to 8) **B)** and regeneration frequencies (positive plantlets/positive callus) (*n* = 2 to 5) **C)** achieved using different vectors when transforming GP. Different letters above the boxes indicate significant differences (*P* < 0.05, one-way analysis of variance (ANOVA)). **D)** Shoot regeneration of callus transformed with different vectors in GP. The formation and shoot regeneration of callus **E)**, as well as transformation efficiencies (positive plantlets/immature embryos) **F)** using control and *HvWUS*  *+*  *HvBBM2* in ZU9, ZU10, Metcalfe, Synergy, and Copeland. **G)** Schematic overview of the vectors used for CRISPR/Cas9 gene editing, including the target information for *HNT1*. **H)** Transformation efficiencies and editing frequencies achieved by U6 and U6WB. **I)** Types of mutations in T_0_ plants generated by U6 and U6WB. **J)** The phenotype of the WT and *hnt1* T1 plants generated by U6 and U6WB, respectively. **K)** Expression patterns of candidate genes identified in DEGs of WB/CK and MEbrown module of WGCNA at 3 stages. The line colors are symbolized as below: black—*HvWUS* and *HvBBM2*; red—genes identified in MEbrown module, GP and Metcalfe; blue—genes identified in MEbrown module and GP; yellow—genes identified in MEbrown module and Metcalfe. The box plots show median (center lines), interquartile range (box limits), and 1.5× interquartile range whisker extensions.

Positive plantlets were identified by green fluorescence from ZsGreen and PCR analyses. Analysis of regeneration frequency (positive plantlets/positive callus) showed that only *HvWUS* + *HvBBM2* was significantly higher (3.08 folds) than control, and the other vectors were not significantly different from the control (Fig. 1C). The insertion of plant growth regulators caused the significant alteration in callus morphology. Compared with the control, callus transformed with *GRF4-GIF1* was loose, watery, and proliferated rapidly, forming solid and large chunks. In contrast, callus transformed with *WUS* and *BBM* grew vigorously but exhibited compact or friable characteristics, similar to the control (Fig. 1D). Several independent replicated experiments were conducted, yielding consistent results. In short, co-expression of *HvWUS* and *HvBBM2* significantly increased both positive rate and regeneration frequency in barley transformation.

The effect of co-expression of *HvWUS* and *HvBBM2* on barley transformation was further evaluated in 5 extremely low-transformable barley genotypes (ZU9, ZU10, Metcalfe, Synergy and Copeland, detail information is provided in Supplementary Table S2). Barley embryos with control vector produced a large amount of nonembryonic callus, as well as a few of embryonic callus, which were almost entirely negative, and did not generate plantlets in multiple repeated experiments (Fig. 1, E and F, Supplementary Fig. S3B and Table S3). However, co-expression of *HvWUS* and *HvBBM2* induced more embryogenic callus with faster proliferation rates, resulting in generation of more positive plantlets (Fig. 1E, Supplementary Figs. S3B and S4, B and D). The transformation efficiencies (positive plantlets/immature embryos) of ZU9, ZU10, Metcalfe, Synergy, and Copeland with co-expression of *HvWUS* and *HvBBM2* differed greatly among the different experiment, ranging from 0.0% to 50.0%, 0.0% to 84.2%, 34.3% to 62.5%, 0.0% to 7.9%, and 0.0% to 9.8%, respectively (Fig. 1F, Supplementary Table S3). Obviously, co-expression of *HvWUS* and *HvBBM2* caused a significant improvement of transformation for these barley genotypes with extremely low transformation efficiency in the previous experiments. However, the transformation system seems unstable, as transformation efficiencies of each genotype differed greatly among the different experiments. After considering all possible contributing factors, we suggest that the most probable cause of the observed difference is the duration of spike storage at 4 °C, which affects embryo viability.

It has been reported that co-expression of *ZmWUS2* and *ZmBBM* in maize can lead to phenotypic abnormalities and sterility (Lowe et al. 2016, 2018). Accordingly, we examined whether co-expression of *HvWUS* and *HvBBM2* has the negative influence on barley growth and development. The Cre/*loxP* system was applied to excise *HvWUS* and *HvBBM2* before plant regeneration (Supplementary Fig. S5), as recommended by Lowe et al. (2016). However, the existing Cre/*loxP* system was inefficient and unstable, since only about 5% of positive plantlets were identified as excised by PCR, and green fluorescence could be still detected in these plants (Supplementary Fig. S4, A and C). We compared 4 groups of plants: (ⅰ) wild-type GP (WT); (ⅱ) plants transformed with the control vector; (ⅲ) plants transformed with *HvWUS* + *HvBBM2* vector; (ⅳ) plants transformed with *HvWUS* + *HvBBM2* vector and successfully excised by Cre recombinase (*HvWUS*  *+*  *HvBBM2*_excised_). The results showed that all transgenic plants transformed with *HvWUS* and *HvBBM2* grew normally and produced grains (Supplementary Fig. S6, A and B). There was no significant difference in spikes per plant and plant height among the 4 groups (Supplementary Fig. S6, D and F). Compared with WT, plants transformed with either the control vector or *HvWUS*  *+*  *HvBBM2* had fewer tillers and slightly thicker stems, while *HvWUS* + *HvBBM2*_excised_ plants were very similar to WT (Supplementary Fig. S6, A–C). According to Murray et al. (2004), the difference in tillers per plant was not caused by *HvWUS* and *HvBBM2*, but affected by other elements on vectors (such as ZsGreen). Moreover, grains per spike were significantly reduced in *HvWUS* + *HvBBM2* plants, but returned to control levels after excision of *HvWUS* and *HvBBM2* (Supplementary Fig. S6E). The change in thousand-grain weight was opposite to grains per spike (Supplementary Fig. S6G). In conclusion, co-expression of *HvWUS* and *HvBBM2* do not affect overall plant growth, but has the distinct influence on grains per spike and thousand-grain weight.

CRISPR/Cas9 gene editing has been widely used in gene functional research and targeted breeding in many crops. In order to determine the possibility of application of the co-expression of *HvWUS* and *HvBBM2* in gene editing system, we inserted *HvWUS* and *HvBBM2* into a CRISPR/Cas9 vector pUB-Cas9-U6 (U6), to generate the vector pUB-Cas9-U6-*HvWUS*  *+*  *HvBBM2* (U6WB). Both constructed vectors contained a guide RNA (gRNA) sequence targeting barley *high number of tillers 1* (*HNT1*) gene (Fig. 1G), which regulates tiller development and leaf width (Ye et al. 2019). All T_0_ plants generated by U6 and U6WB were sequenced, and the results showed that the U6WB vector generated more plantlets without affecting editing efficiency (Fig. 1H, Supplementary Fig. S7). Although significantly more edited plantlets were obtained with U6WB, the diversity of mutation types did not increase correspondingly (Fig. 1I). Moreover, regardless of the vector used, all mutants exhibited a phenotype characterized by more tillers per plant and narrow leaves (Fig. 1J). These results suggest that co-expression of *HvWUS* and *HvBBM2* is favorable for the generation of more transgenic plants without affecting gene editing.

To decipher the regulatory network mediated by *HvWUS* and *HvBBM2* during embryonic callus formation, callus samples were taken at 3 stages, induction, formation and proliferation, cultured on callus induction and selection medium (CISM) for 1, 3, and 7 weeks, respectively. Thus, 6 samples were obtained from calluses of GP transformed with control (CK) and *HvWUS* + *HvBBM2*: G-CK1, G-CK3, G-CK7, G-WB1, G-WB3, and G-WB7. Another 6 samples were taken from calluses of Metcalfe: M-CK1, M-CK3, M-CK7, M-WB1, M-WB3, and M-WB7. For GP, there were only a few differentially expressed genes (DEGs) identified between CK and *HvWUS* + *HvBBM2* at callus induction stage (Supplementary Fig. S8A). With subculture on CISM, the expression levels of *HvWUS* and *HvBBM2* increased, and more DEGs were identified (Fig. 1K, Supplementary Fig. S8A). A similar trend was also found for Metcalfe. Furthermore, in the calluses with highly expressed *HvWUS* and *HvBBM2* at callus proliferation stage, DEGs were significantly enriched in “Transcription factors”, “Plant hormone signal transduction”, and other pathways (Supplementary Fig. S8B). Additionally, WGCNA identified genes exhibiting highly synergistic changes with expression levels of *HvWUS* and *HvBBM2*. The MEbrown module, which includes *HvWUS* and *HvBBM2*, was selected as the target module (Supplementary Fig. S8C). The genes shared between DEGs in GP and Metcalfe, along with the genes in the MEbrown module, were picked out (Supplementary Fig. S8D), and some were further identified as a series of candidate genes according to their annotations (Fig. 1K, Supplementary Table S4). Taken together, we assume that transcription factors, including MYB91, MYB94, AGL22, AGL52, MADS, and NAC6, as well as genes related to plant hormones, including LAX2, GASA4, IAA3, IAA8, ARP/DRM, ARP/DRM-2, ARF3, and ARF3-2 may be involved in barley transformation enhanced by *HvWUS* and *HvBBM2*.

In conclusion, this study demonstrates that co-expression of *HvWUS* and *HvBBM2* significantly enhances generation of fertile plantlets in both GP and previously-proven extremely low-transformable barley genotypes. Furthermore, genome editing was successfully achieved by integrating *HvWUS* and *HvBBM2* into the CRISPR/Cas9 system. Notably, the obvious improvement of barley transformation efficiency achieved in this study provides an approach for advancing functional genome research and breeding in barley.

## Supplementary Material

kiaf494_Supplementary_Data

## Data Availability

The data underlying this article are available in the article and its online supplementary material.

## Supplementary data

The following materials are available in the online version of this article.

**Supplementary Figure S1.** Identification of WUS and BBM in barley.

**Supplementary Figure S2.** Sequence alignment and domain analysis in barley, wheat, rice, maize, and Arabidopsis.

**Supplementary Figure S3.** Identification of positive callus through green fluorescence from ZsGreen.

**Supplementary Figure S4.** Positive verification of transgenic T1 plants.

**Supplementary Figure S5.** Diagram of the Cre/*loxP* system.

**Supplementary Figure S6.** Phenotype of wild-type GP as well as transgenic plants harboring CK, *HvWUS*  *+*  *HvBBM2*, or *HvWUS*  *+*  *HvBBM2*_excised_.

**Supplementary Figure S7.** Callus and regenerated plantlets transformed using pUB-Cas9-U6 (i.e. U6) or pUB-Cas9-U6-*HvWUS*  *+*  *HvBBM2* (i.e. U6WB).

**Supplementary Figure S8.** Transcriptome analysis of callus transformed with CK or *HvWUS*  *+*  *HvBBM2* in the GP (G) or Metcalfe (M) background at 3 stages of culture.

**Supplementary Table S1.** Identification of plant growth regulators in barley.

**Supplementary Table S2.** The detail information of ZU9, ZU10, Metcalfe, Synergy, and Copeland.

**Supplementary Table S3.** Summary of barley immature embryo transformation in ZU9, ZU10, Metcalfe, Synergy, and Copeland.

**Supplementary Table S4.** Detailed information on the candidate genes.
