# Supplementary material for: Co-expression of growth regulator genes HvWUS and HvBBM2 enhances barley transformation efficiency
Source: Plant Physiol. 2025 Oct 28;199(2):kiaf494. doi: 10.1093/plphys/kiaf494 (PMC12559888; doi:10.1093/plphys/kiaf494)
Supplement: kiaf494_Supplementary_Data [file kiaf494_supplementary_data.zip › PLPHYS-2025-1045R2_Supplementary Figures, Tables, Methods.pdf]

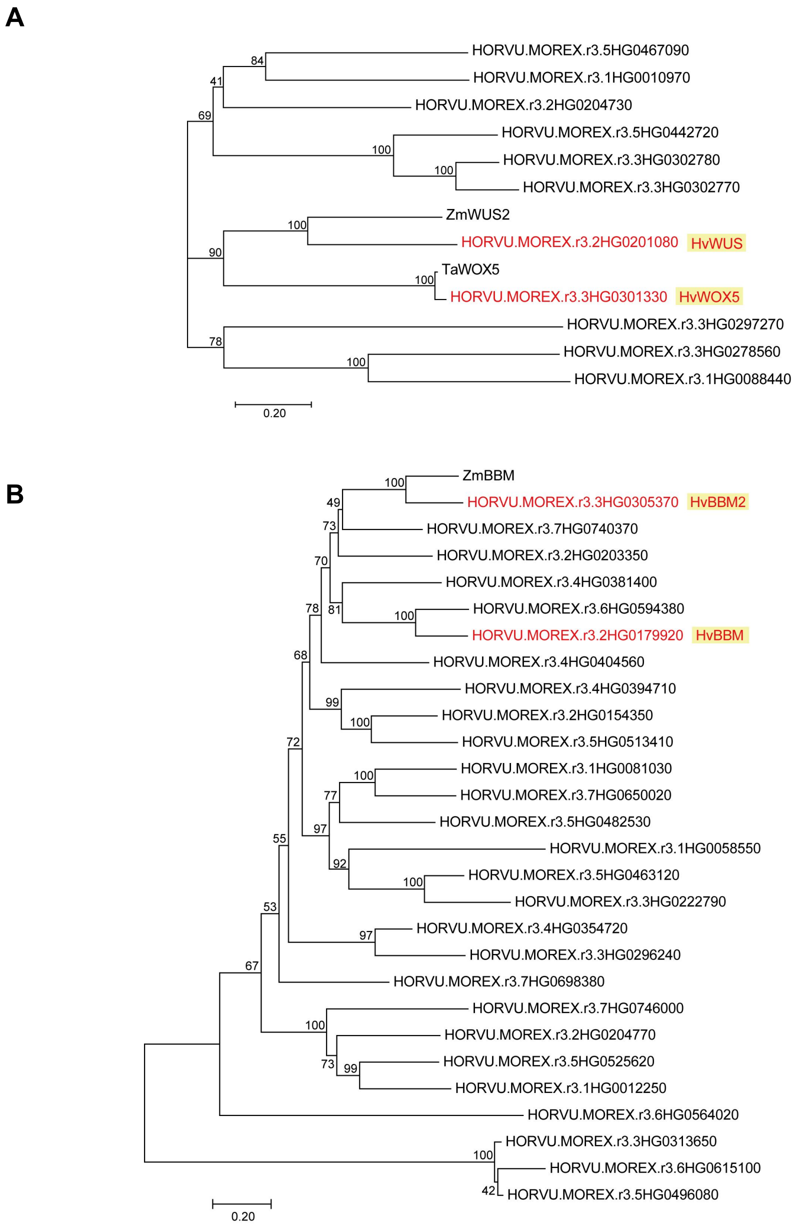

Supplementary Figure S1. Identification of WUS and BBM in barley. Phylogenetic relationship of WUS **A)**, and BBM **B)**. Phylogenetic tree analyses were performed using MEGA 7.0 and the maximum likelihood method with the default parameters. The numbers on the branching points represent the bootstrap values. The scale bar indicates a length of 0.20 substitution per site.

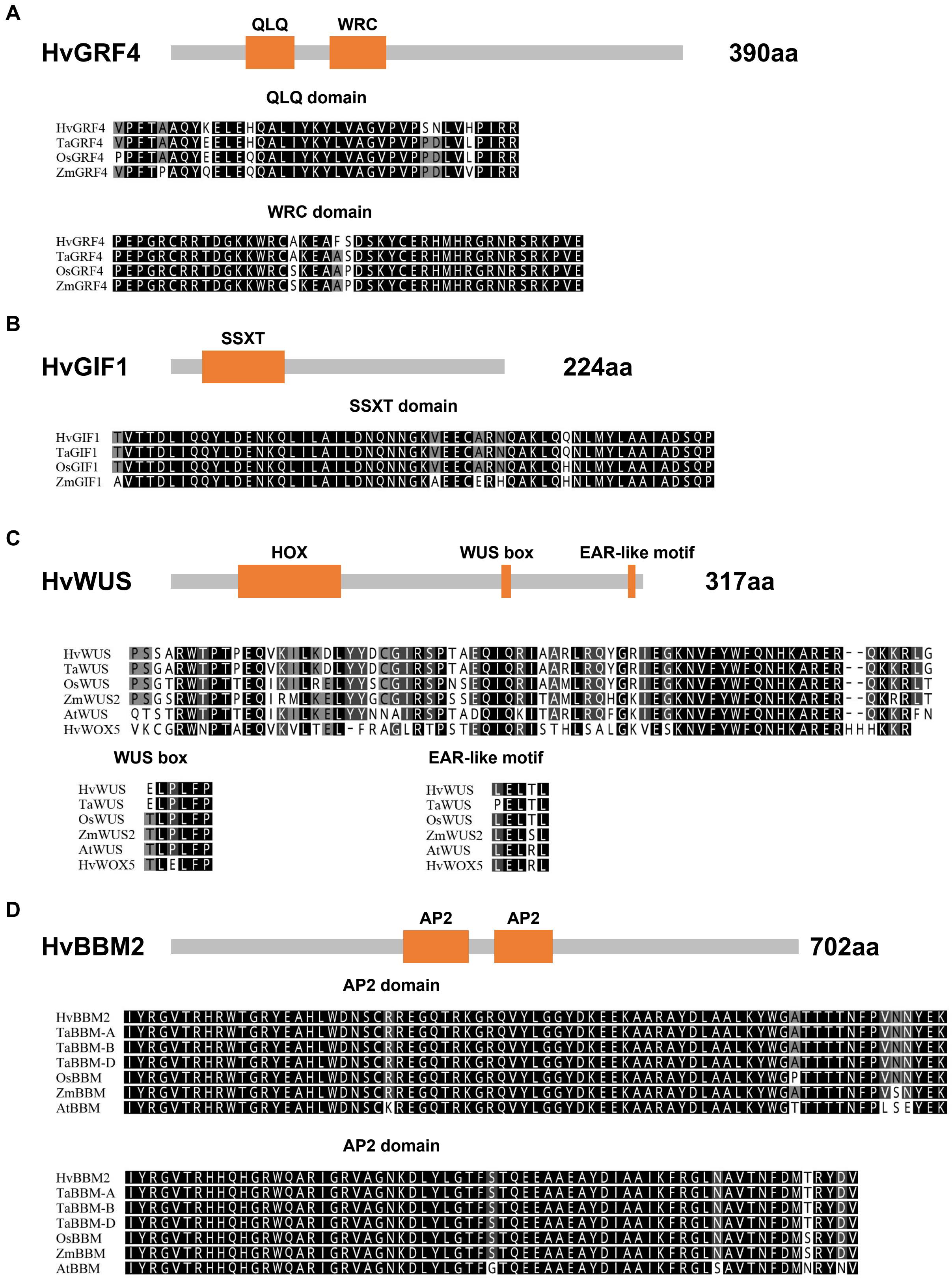

Supplementary Figure S2. Sequence alignment and domain analysis in barley, wheat, rice, maize, and Arabidopsis. **A)** GRF4. **B)** GIF1. **C)** WUS. **D)** BBM2.

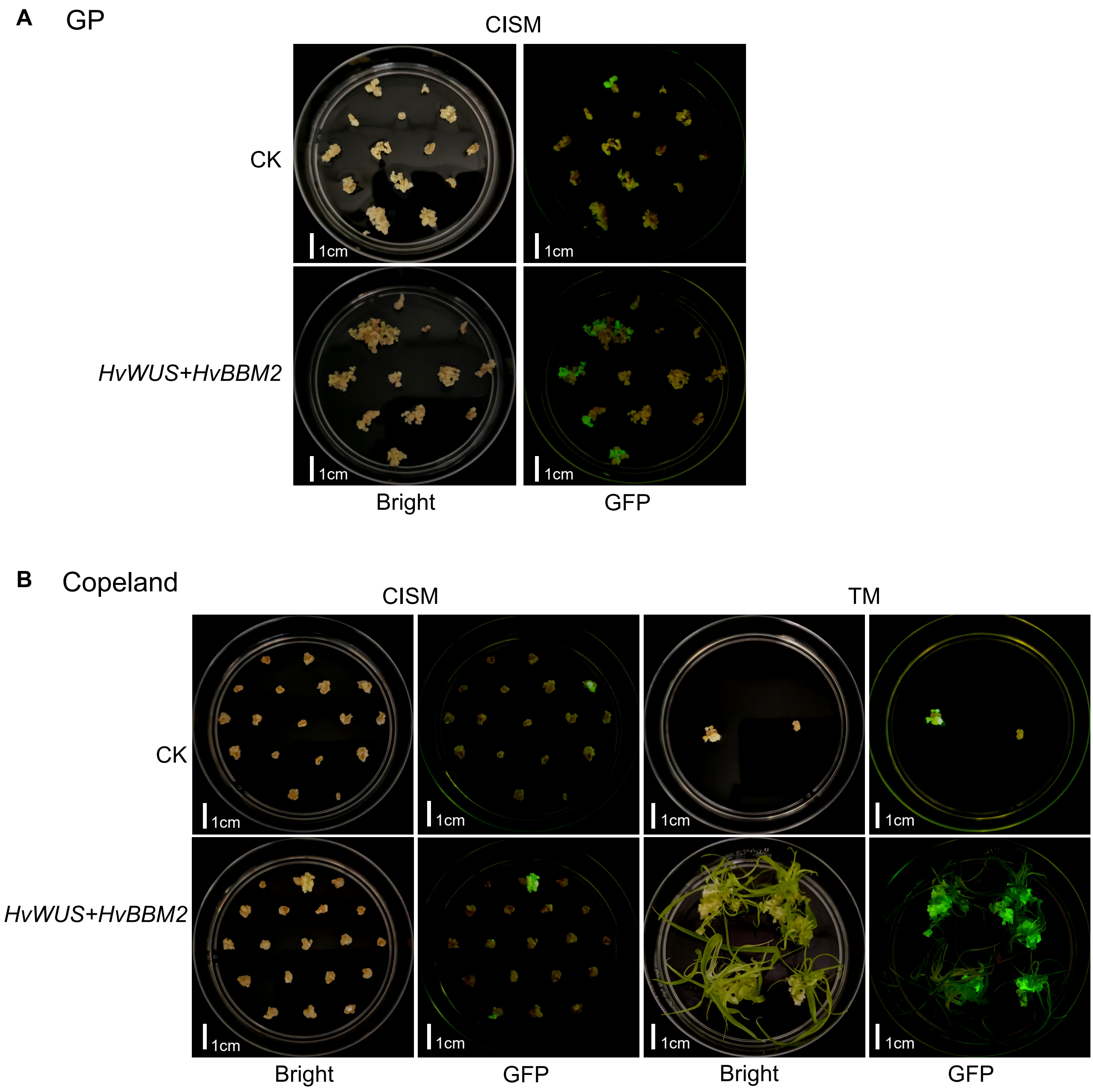

Supplementary Figure S3. Identification of positive callus through green fluorescence from ZsGreen. Fluorescence of positive callus transformed by control vector (CK) and *HvWUS+HvBBM2* in GP **A)** and Copeland **B)** on callus induction and selection medium (CISM), and transition medium (TM). The bright images for Copeland **B)** are the same as those shown in Figure 1E.

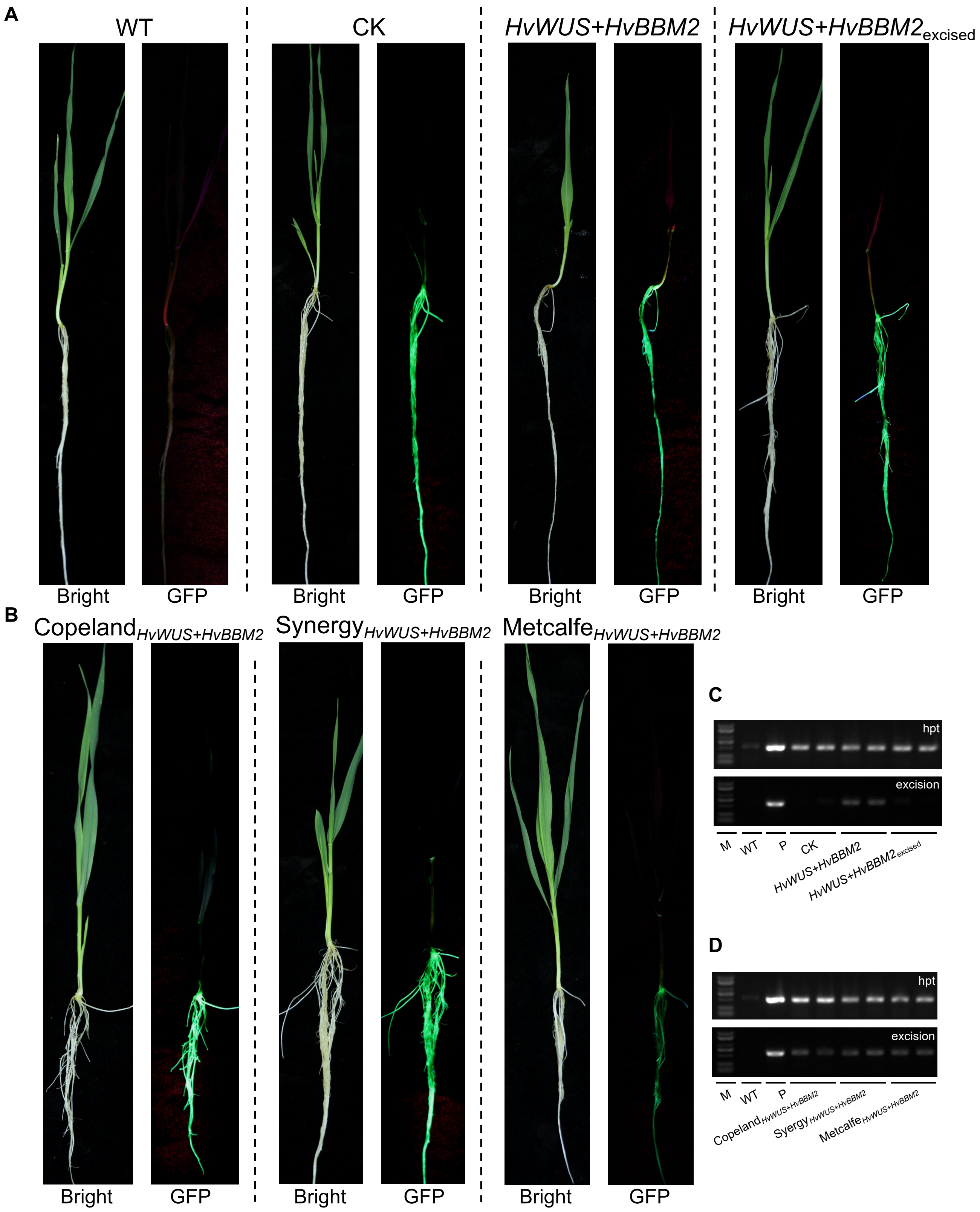

Supplementary Figure S4. Positive verification of transgenic T1 plants. **A)** Fluorescence of transgenic plants in GP. **B)** Fluorescence of transgenic plants in Copeland, Synergy and Metcalfe. **C)** PCR amplification for transgenic plants in GP. **D)** PCR amplification for transgenic plants in Copeland, Synergy and Metcalfe. P is the plasmid with pCAMBIA1300-*HvWUS*+*HvBBM2* vector.

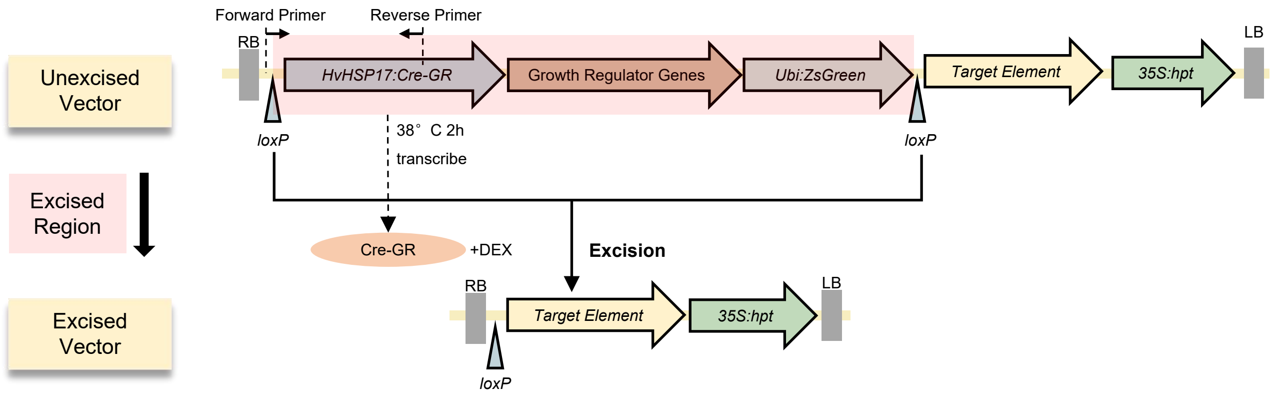

Supplementary Figure S5. Diagram of the Cre/*loxP* system.

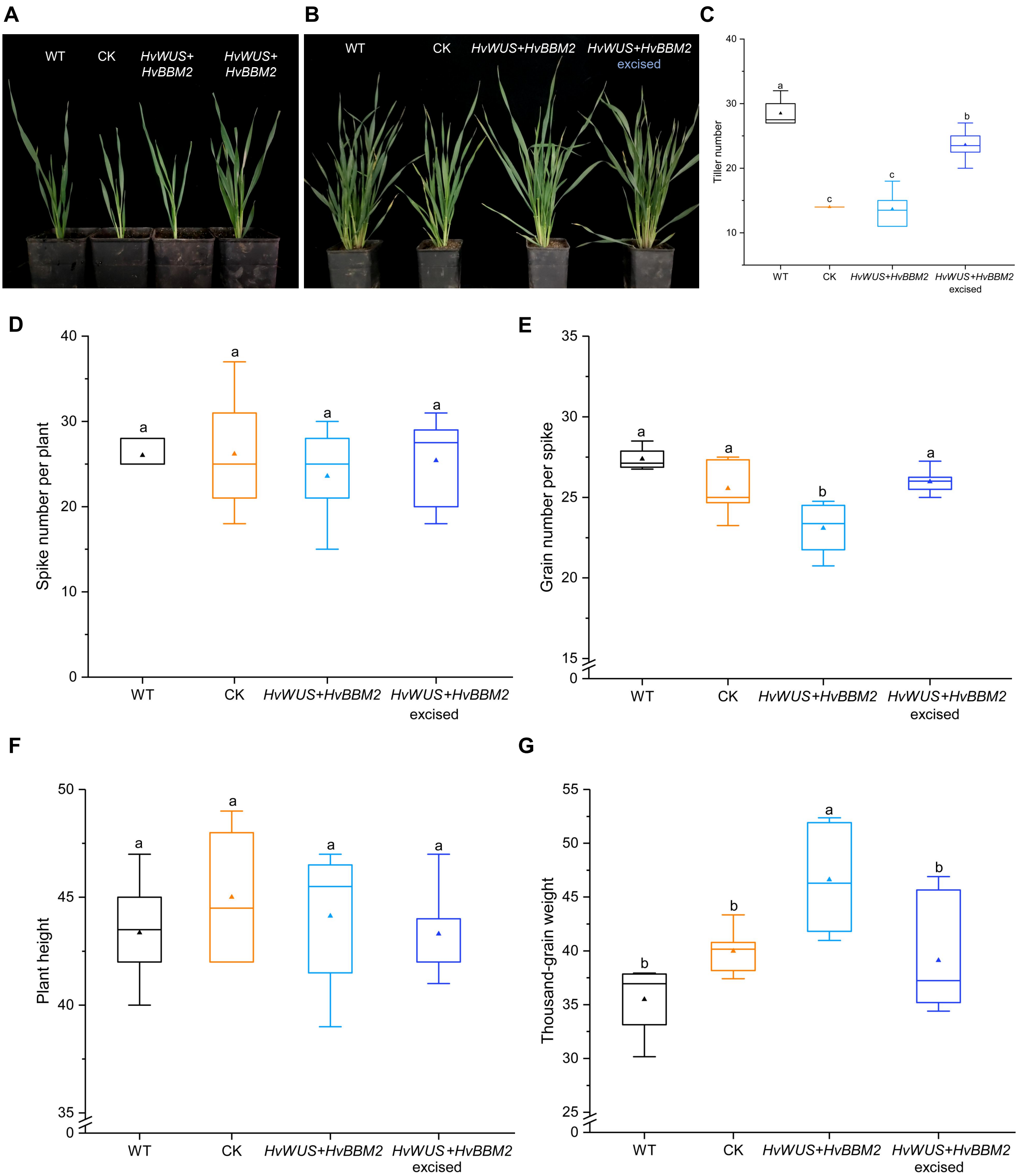

Supplementary Figure S6. Phenotype of wild-type GP as well as transgenic plants harboring CK, *HvWUS+HvBBM2*, or *HvWUS+HvBBM2*$_{\text{excised}}$. **A)** Seedling appearance. **B)** Tillering stage appearance. **C)** Tiller number (*n* = 2 to 8). **D)** Spike number per plant (*n* = 3 to 10). **E)** Grain number per spike (*n* = 4 to 7). **F)** Plant height (cm) (*n* = 6 to 14). **G)** Thousand-grain weight (g) (*n* = 4 to 7). Different letters indicate statistically significant differences at $p < 0.05$ based on one-way ANOVA. The box plots show median (center lines), interquartile range (box limits), and 1.5× interquartile range whisker extensions, while triangles indicate arithmetic means.

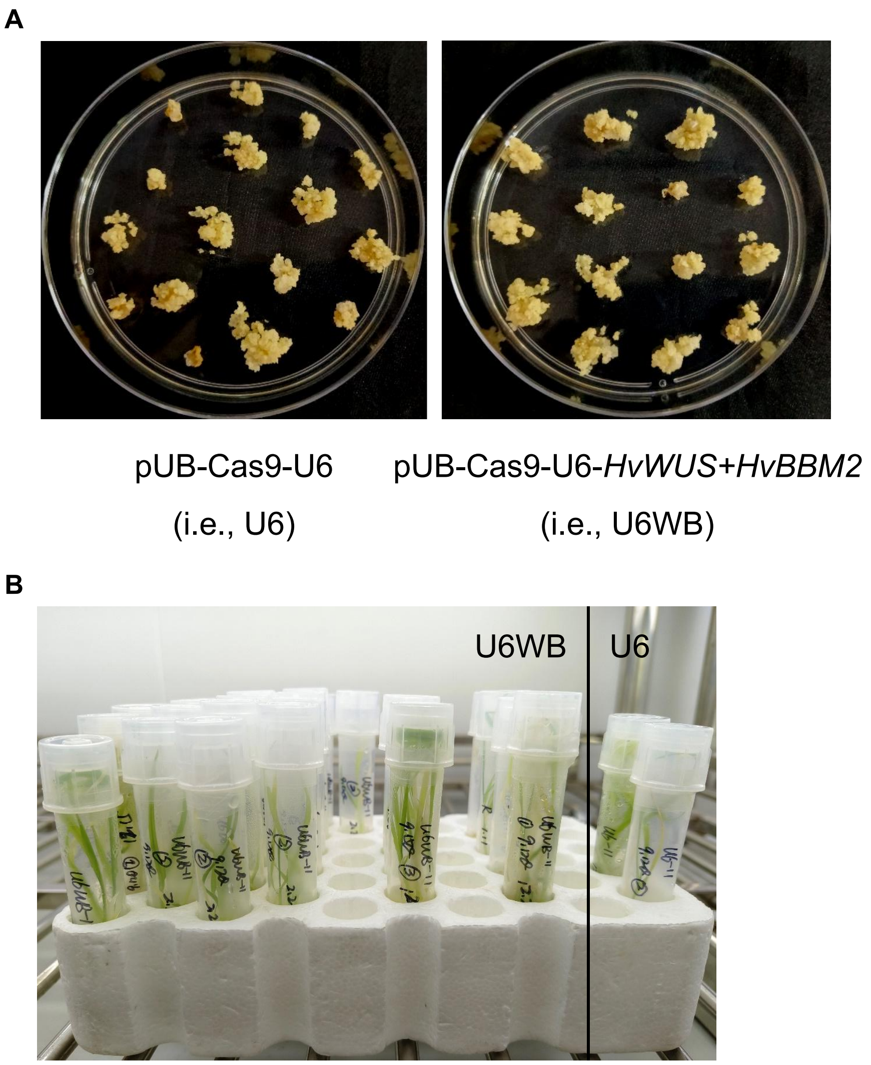

Supplementary Figure S7. Callus and regenerated plantlets transformed using pUB-Cas9-U6 (i.e., U6) or pUB-Cas9-U6-*HvWUS*+*HvBBM2* (i.e., U6WB). **A)** Callus. **B)** Regenerated plantlets.

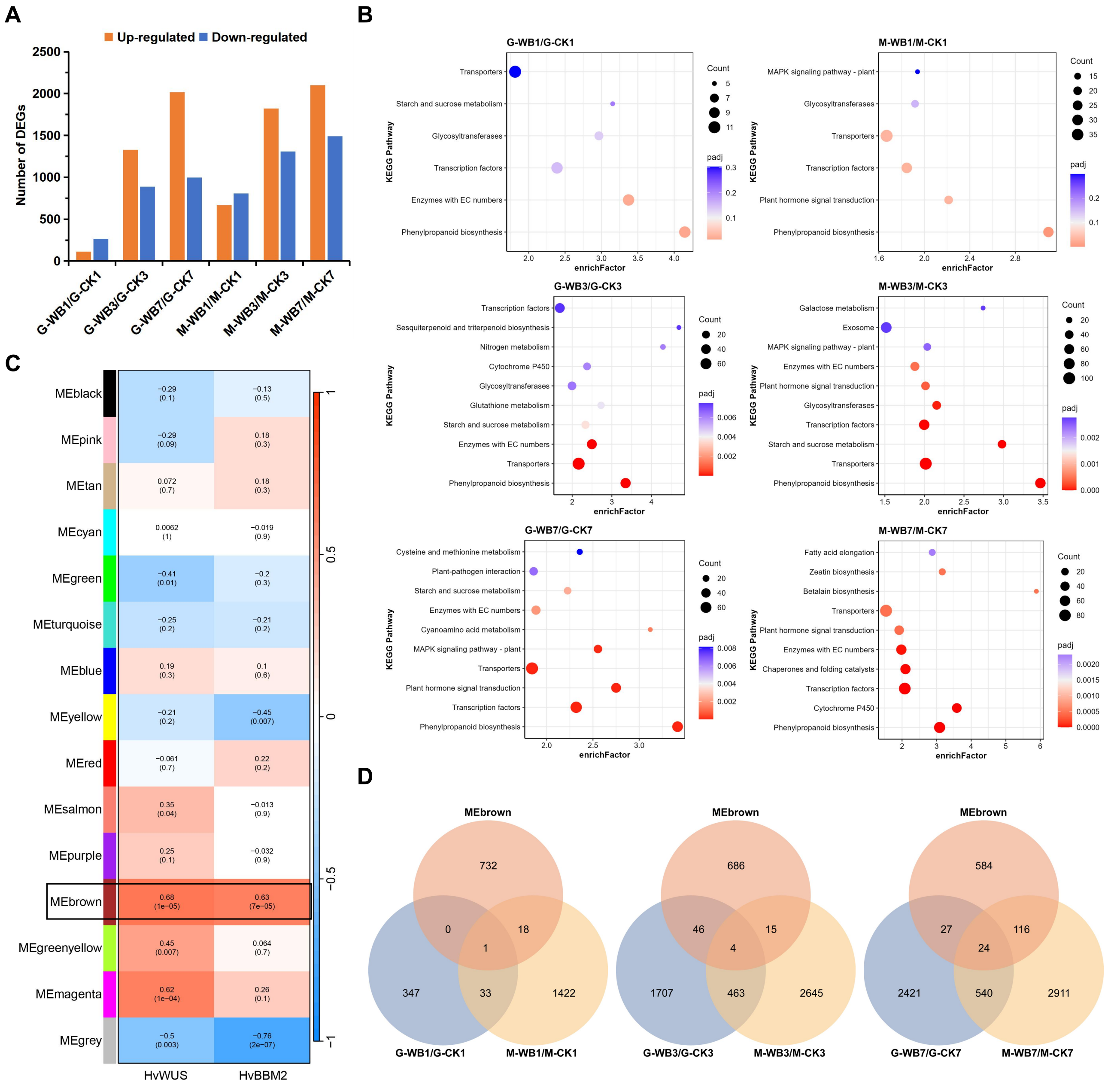

Supplementary Figure S8. Transcriptome analysis of callus transformed with CK or *HvWUS+HvBBM2* in the GP (G) or Metcalfe (M) background at three stages of culture. **A)** The number of differentially expressed gens (DEGs) between callus transformed with CK and *HvWUS+HvBBM2* (WB). **B)** Kyoto Encyclopedia of Genes and Genomes (KEGG) pathways of DEGs. **C)** The relationship between the modules identified by Weighted Gene Co-Expression Network Analysis (WGCNA) and the expression levels of *HvWUS* and *HvBBM2*. **D)** Venn diagrams of genes identified in MEbrown module of WGCNA and DEGs of WB/CK in GP and Metcalfe at 1 (left), 3 (middle), and 7 (right) weeks of culture.

Supplementary Table S1. Identification of plant growth regulators in barley.

Gene Name	Gene ID or GenBank ID
HvWUS	HORVU.MOREX.r3.2HG0201080
HvBBM2	HORVU.MOREX.r3.3HG0305370
HvWOX5	HORVU.MOREX.r3.3HG0301330
HvGRF4	HORVU.MOREX.r3.6HG0606810
HvGIF1	HORVU.MOREX.r3.4HG0339920
ZmWUS2	ABW43772.1
ZmBBM	CAJ29869.1
TaGRF4	TraesCS6A01G269600
TaGIF1	TraesCS4A01G250600

Supplementary Table S2. The detail information of ZU9, ZU10, Metcalfe, Synergy, and Copeland.

Variety	Full Name	Row Type	Country of Origin	Domestication Status	Growth Habit	Use
ZU9	Zhejing University NO.9	2	China	Cultivar	Semi-winter	Malting & Feeding
ZU10	Zhejing University NO.10	2	China	Cultivar	Semi-winter	Feeding
Metcalfe	AC Metcalfe	2	Canada	Cultivar	Spring	Malting
Synergy	AAC Synergy	2	Canada	Cultivar	Spring	Malting
Copeland	CDC Copeland	2	Canada	Cultivar	Spring	Malting

Supplementary Table S3. Summary of barley immature embryo transformation in ZU9, ZU10, Metcalfe, Synergy, and Copeland.

Variety	Experiment	Vector	No. of immature embryos	No. of induced calli	% of induced calli	No. of GFP positive calli	% of GFP positive calli	No. of positive plantlets	Freq. of positive plantlets	Transformation efficiency (%)
ZU9	1	CK	135	100	74.1	6	6.0	0	0.00	0.0
		HvWUS+HvBBM2	70	60	85.7	27	45.0	35	1.30	50.0
	2	CK	36	21	58.3	0	0.0	0	/	0
		HvWUS+HvBBM2	31	30	96.8	1	3.3	0	0.00	0.0
	3	CK	248	234	94.4	1	0.4	0	0.00	0.0
		HvWUS+HvBBM2	322	313	97.2	14	4.5	2	0.14	0.6
ZU10	1	CK	159	135	84.9	11	8.1	0	0.00	0.0
		HvWUS+HvBBM2	114	103	90.4	35	34.0	96	2.74	84.2
	2	CK	71	55	77.5	0	0.0	0	/	0
		HvWUS+HvBBM2	66	60	90.9	5	8.3	3	0.60	4.5
	3	CK	127	116	91.3	3	2.6	0	0.00	0.0
		HvWUS+HvBBM2	85	76	89.4	13	17.1	0	0.00	0.0
Metcalfe	1	CK	43	42	97.7	1	2.4	0	0.00	0.0
		HvWUS+HvBBM2	80	74	92.5	9	12.2	50	5.56	62.5
	2	CK	79	62	78.5	0	0.0	0	/	0
		HvWUS+HvBBM2	99	80	80.8	25	31.3	34	1.36	34.3

Synergy	1	CK	163	78	47.9	0	0.0	0	/	0
		HvWUS+HvBBM2	248	110	44.4	0	0.0	0	/	0
	2	CK	67	47	70.1	1	2.1	0	0.00	0.0
		HvWUS+HvBBM2	101	90	89.1	10	11.1	8	0.80	7.9
	3	CK	179	144	80.4	1	0.7	0	0.00	0.0
		HvWUS+HvBBM2	162	91	56.2	14	15.4	8	0.57	4.9
Copeland	1	CK	270	177	65.6	3	1.7	0	0.00	0.0
		HvWUS+HvBBM2	164	139	84.8	16	11.5	16	1.00	9.8
	2	CK	137	75	54.7	0	0.0	0	/	0
		HvWUS+HvBBM2	247	154	62.3	9	5.8	24	2.67	9.7
	3	CK	188	124	66.0	0	0.0	0	/	0
		HvWUS+HvBBM2	282	152	53.9	7	4.6	11	1.57	3.9

Supplementary Table S4. Detailed information on the candidate genes.

gene name	gene ID	annotation
MYB91	HORVU.MOREX.r3.5HG0441760	Myb transcription factor
MADS	HORVU.MOREX.r3.3HG0286170	MADS-box transcription factor
LAX2	HORVU.MOREX.r3.1HG0017310	Auxin influx transporter
WAT1	HORVU.MOREX.r3.3HG0219290	WAT1-related protein
ZPR3	HORVU.MOREX.r3.2HG0166000	Homeobox-leucine zipper protein HOX9 putative
AGL22	HORVU.MOREX.r3.4HG0406150	MADS box transcription factor
HSP70	HORVU.MOREX.r3.4HG0387610	70 kDa heat shock protein
CKI	HORVU.MOREX.r3.5HG0526800	cyclin-dependent kinase inhibitor
LEA6	HORVU.MOREX.r3.1HG0061780	Late embryogenesis abundant protein
AGL52	HORVU.MOREX.r3.5HG0428810	AGAMOUS-like 52
GASA4	HORVU.MOREX.r3.7HG0670790	Gibberellin-regulated family protein
NAC6	HORVU.MOREX.r3.2HG0111410	NAC domain protein
IAA3	HORVU.MOREX.r3.5HG0433440	Auxin-responsive protein
ARP/DRM	HORVU.MOREX.r3.4HG0376000	Auxin-repressed/dormancy-associated protein
ARP/DRM-2	HORVU.MOREX.r3.5HG0480830	Auxin repressed/dormancy associated protein
MYB94	HORVU.MOREX.r3.2HG0122400	Myb factor
IAA8	HORVU.MOREX.r3.1HG0086460	Auxin-responsive protein
ARF3	HORVU.MOREX.r3.3HG0289370	Auxin response factor
ARF3-2	HORVU.MOREX.r3.1HG0087670	Auxin response factor

Supplementary materials and methods

Plant materials and growing conditions

Barley cultivars Golden Promise (GP), ZU9, ZU10, Metcalfe, Synergy and Copeland, along with transgenic plants, were grown under controlled environmental conditions in a growth chamber set to a 16 h/8 h light/dark photoperiod, 20 ± 2°C temperature, and 60–70% relative humidity. When the plants reached the grain-filling stage and embryo length was approximately 2–3 mm, spikes were harvested and stored at 4°C until subsequent embryo isolation and tissue culture procedures. Due to limitations in daily workload, embryo isolation and repeated experiments were conducted in batches. For ZU9, ZU10, Metcalfe, Synergy, and Copeland genotypes used in the validation of WUS2+BBM2, the first trial utilized freshly isolated immature embryos without cold storage. The second trial used embryos stored at 4°C for 24–36 hours, and the third trial used embryos stored at 4°C for 36–96 hours.

Gene identification and cloning

The amino acid sequences of TaGRF4, TaGIF1, ZmWUS2 and ZmBBM were retrieved from the studies by Debernardi et al. (2020) and Lowe et al. (2016). These sequences were used for BLASTP searches against the barley genome, with an e-value threshold of <1e^{-10}. HORVU.MOREX.r3.6HG0606810 and HORVU.MOREX.r3.4HG0339920 were identified as HvGRF4 and HvGIF1, due to their highest similarity to TaGRF4 and TaGIF1. Based on phylogenetic analyses using MEGA 7.0 with the default parameters, HORVU.MOREX.r3.3HG0301330 reported by Suo et al. (2021) was renamed as HvWOX5, while HvWUS (HORVU.MOREX.r3.2HG0201080) and HvBBM2 (HORVU.MOREX.r3.3HG0305370) were deemed homologous to ZmWUS2 and ZmBBM, respectively (Supplementary Fig. S1). HvGRF4, HvGIF1, HvBBM2 and HvWOX5 were cloned from cDNA of GP, while TaGRF4, TaGIF1, ZmWUS2, ZmBBM and HvWUS were synthesized by the company (GenScript, USA).

Vector construction

ZsGreen (Lowe et al. 2016) was cloned into the pCAMBIA1300 vector along with Ubi promoter and Nos terminator to create pCAMBIA1300-CK. *HvGRF4-GIF1* and *HvGRF4-GIF1* chimeras were inserted into pCAMBIA1300-CK to generate pCAMBIA1300-*HvGRF4-GIF1* and pCAMBIA1300-*TaGRF4-GIF1*, respectively. To integrate Cre/*loxP* system into pCAMBIA1300, pCAMBIA1300-Cre was constructed with the following three expression cassettes: (1) Cre recombinase (Lowe et al. 2016) fused with glucocorticoid receptor (GR) (Zhai and Xu 2020), driven by the HvHSP17 promoter (Freeman et al. 2011), (2) two *loxP* sites, (3) ZsGreen with the Ubi promoter. *HvWUS* with AXIG1 promoter and *HvBBM2* with PLTP promoter (Lowe et al. 2018) were cloned into pCAMBIA1300-Cre to produce pCAMBIA1300-*HvWUS+HvBBM2*. Similarly pCAMBIA1300-*ZmWUS2+ZmBBM* and pCAMBIA1300-*HvWOX5+HvBBM2*, were separately inserted with *ZmWUS2* and *ZmBBM*, as well as

HvWOX5 and *HvBBM2*, instead of *HvWUS* and *HvBBM2*. *HvWUS* and *HvBBM2* were additionally combined with CRISPR/Cas9 vector pUB-Cas9-U6 to generate pUB-Cas9-U6-*HvWUS+HvBBM2*, as shown in Fig. 1G.

Barley transformation

Barley transformation was conducted using a modified protocol based on Harwood (2014) as follows: (1) Intact immature seeds were surface-sterilized with 70% ethanol for 30 seconds, washed three times with sterilized water, stirred in 50% (v/v) sodium hypochlorite for 4 min, and washed at least four more times with sterile water. (2) Embryos were isolated from the seeds with the removal of embryonic axis, and cultured in the dark for 3 days on callus induction medium (CIM). CIM media was composed of 4.3 g/L MS salt base, 30 g/L maltose, 1.0 g/L casein hydrolysate, 690 mg/L proline, 350 mg/L myoinositol, 1.0 mg/L thiamine HCl, 1.25 mg/L $CuSO_4 \cdot 5H_2O$, pH = 5.8 (using 4M NaOH), 3.5 g/L phytagel and 2.5 mg/L dicamba. (3) Embryos was infected using *Agrobacterium* strain AGL1 with an OD600 of 0.8–0.9, and a new CIM media was used for three-days co-cultivation after incubation for 30 minutes. (4) Embryos were transferred to callus induction and selection medium (CISM), which contained CIM supplemented with 50 mg/L hygromycin and 160 mg/L timentin, and cultured in a two-week cycle. (5) After two months of cultivation, a hand-held lamp (LUYOR-3415RG, China) with blue light was used to distinguish positive callus with green fluorescence from embryos for further culture. (6) Positive calluses were transferred into fresh transition medium (TM) under light every two weeks for shoot induction. TM consisted of 2.7 g/L MS salt base without NH_4NO_3, 165 mg/L NH_4NO_3, 20 g/L maltose, 750 mg/L glutamine, 100 mg/L myoinositol, 0.4 mg/L thiamine HCl, 1.25 mg/L $CuSO_4 \cdot 5H_2O$, pH = 5.8 (using 4M NaOH), 3.5 g/L phytagel, 2.5 mg/L 2,4-dichlorophenoxy acetic acid (2,4-D), 0.1 mg/L 6-benzylaminopurine (6-BA), 50 mg/L hygromycin and 160 mg/L timentin. (7) For Cre/*loxP* system, calluses were transferred to TM media supplemented with 10 μM dexamethasone (Zhai and Xu 2020), and heated at 38°C for two hours (Freeman et al. 2011) to activate Cre recombinase expression. (8) Shoots were placed on TM without 2,4-D and 6-BA, until they reached a height of 3 cm. (9) Elongated shoots were placed in tubes containing 15 mL CISM without dicamba to induce root development. (10) Plantlets with vigorous roots were transferred to hydroponic nutrient solution for establishment. Successfully transformed plantlets with green fluorescence under blue light were further confirmed via PCR analysis targeting the *hpt* gene. (11) Positive plantlets were planted in soil and grown until harvest.

Transgenic events and genome editing events identification

PCR analyses were employed to confirm successful transformation and excision. Transformed plants were identified by amplifying a 542-bp *hpt* gene coding region using primers 5'-TAAATAGCTGCGCCGATGGT-3' and 5'-GGCGACCTCGTATTGGGAAT-3'. To determine excision within the *loxP* site (Supplementary Fig. S5), a 572-bp region spanning both inside and outside the *loxP*

site was amplified using primers 5'-GGTTTTCCCAGTCACGACGT-3' and 5'-TCTCGAAGACTCTCCAACCG-3'. Genome editing events were identified using primers 5'-TGTCCACTACGCCTGTCAAC-3' and 5'-CACACCAAACACCTCCTGGA-3', which amplified PCR products encompassing editing targets of *HNT1*.

Agronomic properties analysis

Tiller numbers were counted before jointing. Plant height was measured from the soil surface to the top of the panicle (excluding awns) before harvest. Effective panicles were identified as those with plump grains and counted. The average number of plump grains per panicle was calculated by randomly selecting four panicles from each plant. Thousand-grain weight was calculated as 10 times the weight of 100 grains.

Transcriptome sequencing and data analysis

RNA extraction was performed using Plant RNA Extraction Kit (Takara, Japan). RNA integrity, cDNA library construction, and RNA sequencing were conducted by Zhejiang University Analysis Center of Agrobiology and Environmental Sciences (Hangzhou, China). Clean reads were obtained by filtering raw reads to remove adapters, poly-N and low-quality reads using Fastp software (Chen 2023). Clean reads were mapped to the reference genome of barley cv. Morex V3 2020 (https://galaxy-web.ipk-gatersleben.de/) using HISAT2 software (Kim et al. 2015). FeatureCounts was used to count the reads numbers mapped to each gene (Liao et al. 2014), and then gene expression levels were estimated with TPM (Transcripts Per Kilobase of exon model per Million mapped reads). Differentially expressed genes (DEGs) were identified by DESeq2 (Love et al. 2014) with the selection criteria of |Log2Fold Change| > 1 and padj < 0.05. Kyoto Encyclopedia of Genes and Genomes (KEGG) enrichment analysis was conducted by TBtools software (Chen et al. 2023) based on the annotations of eggNOG-Mapper (http://eggnog-mapper.embl.de/). Gene coexpression network construction and module identification were conducted using the R package WGCNA (version 1.72-1) (Langfelder and Horvath 2008).

References

Chen C, Wu Y, Li J, Wang X, Zeng Z, Xu J, Liu, Y, Feng J, Chen H, He Y, Xia R. TBtools-II: a “one for all, all for one” bioinformatics platform for biological big-data mining. *Mol. Plant* 2023:16(11):1733–1742. https://doi.org/10.1016/j.molp.2023.09.010

Chen SF. Ultrafast one-pass FASTQ data preprocessing, quality control, and deduplication using fastp. *iMeta* 2023:2(2):e107. https://doi.org/10.1002/imt2.107

Debernardi JM, Tricoli DM, Ercoli MF, Hayta S, Ronald P, Palatnik JF, Dubcovsky J. A GRF-GIF

chimeric protein improves the regeneration efficiency of transgenic plants. *Nat. Biotechnol.* 2020:38:1274–1279. https://doi.org/10.1038/s41587-020-0703-0

Freeman J, Sparks CA, West J, Shewry PR, Jones HD. Temporal and spatial control of transgene expression using a heat-inducible promoter in transgenic wheat. *Plant Biotechnol. J.* 2011:9(7):788–796. https://doi.org/10.1111/j.1467-7652.2011.00588.x

Harwood WA. A protocol for high-throughput *Agrobacterium*-mediated barley transformation. In: Henry RJ, Furtado A, editors. *Cereal Genomics: Methods and Protocols*. Totowa (NJ): Humana Press; 2014. p. 251–260.

Kim D, Langmead B, Salzberg SL. HISAT: a fast spliced aligner with low memory requirements. *Nat. Methods* 2015:12:357–360. https://doi.org/10.1038/nmeth.3317

Langfelder, P., Horvath, S. WGCNA: an R package for weighted correlation network analysis. *BMC Bioinform.* 2008:9:559. https://doi.org/10.1186/1471-2105-9-559

Liao Y, Smyth GK, Shi W. featureCounts: an efficient general purpose program for assigning sequence reads to genomic features. *Bioinformatics* 2014:30(7):923–930. https://doi.org/10.1093/bioinformatics/btt656

Love MI, Huber W, Anders S. Moderated estimation of fold change and dispersion for RNA-seq data with DESeq2. *Genome Biol.* 2014:15:550. https://doi.org/10.1186/s13059-014-0550-8

Lowe K, Wu E, Wang N, Hoerster G, Hastings C, Cho MJ, Scelonge C, Lenderts B, Chamberlin M, Cushatt J, *et al*. Morphogenic regulators *Baby boom* and *Wuschel* improve monocot transformation. *Plant Cell* 2016:28(9):1998–2015. https://doi.org/10.1105/tpc.16.00124

Lowe K, La Rota M, Hoerster G, Hastings C, Wang N, Chamberlin M, Wu E, Jones T, Gordon-Kamm W. Rapid genotype "independent" *Zea Mays* L. (maize) transformation via direct somatic embryogenesis. *In Vitro Cell. Dev. Biol.-Plant* 2018:54:240–252. https://doi.org/10.1007/s11627-018-9905-2

Suo JQ, Zhou CL, Zeng ZH, Li XP, Bian HW, Wang JH, Zhu MY, Han N. Identification of regulatory factors promoting embryogenic callus formation in barley through transcriptome analysis. *BMC Plant Biol.* 2021:21:145. https://doi.org/10.1186/s12870-021-02922-w

Wang K, Shi L, Liang XN, Zhao P, Wang WX, Liu JX, Chang YA, Hiei Y, Yanagihara C, Du LP, *et al.* The gene *TaWOX5* overcomes genotype dependency in wheat genetic transformation. *Nat. Plants* 2022:8:110–117. https://doi.org/10.1038/s41477-021-01085-8

Zhai N, Xu L. Cre/Lox-based analysis of cell lineage during root formation and regeneration in *Arabidopsis*. *aBIOTECH* 2020:1:153–156. https://doi.org/10.1007/s42994-020-00025-y
