# Supplementary material for: Co-expression of growth regulator genes HvWUS and HvBBM2 enhances barley transformation efficiency
Source: Plant Physiol. 2025 Oct 28;199(2):kiaf494. doi: 10.1093/plphys/kiaf494 (PMC12559888; doi:10.1093/plphys/kiaf494)
Supplement: kiaf494_Supplementary_Data [file kiaf494_supplementary_data.zip › Supplementary Data.pdf]

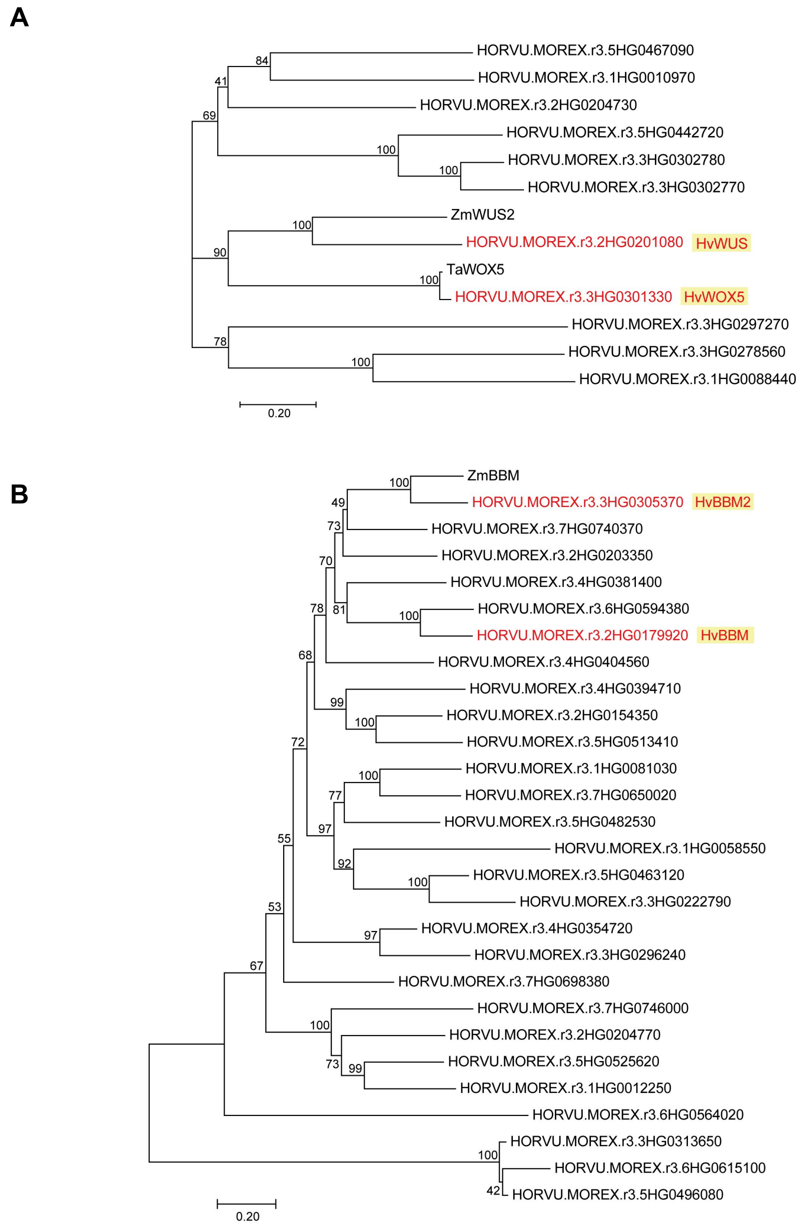

Supplementary Figure S1. Identification of WUS and BBM in barley. Phylogenetic relationship of WUS **A)**, and BBM **B)**. Phylogenetic tree analyses were performed using MEGA 7.0 and the maximum likelihood method with the default parameters. The numbers on the branching points represent the bootstrap values. The scale bar indicates a length of 0.20 substitution per site.

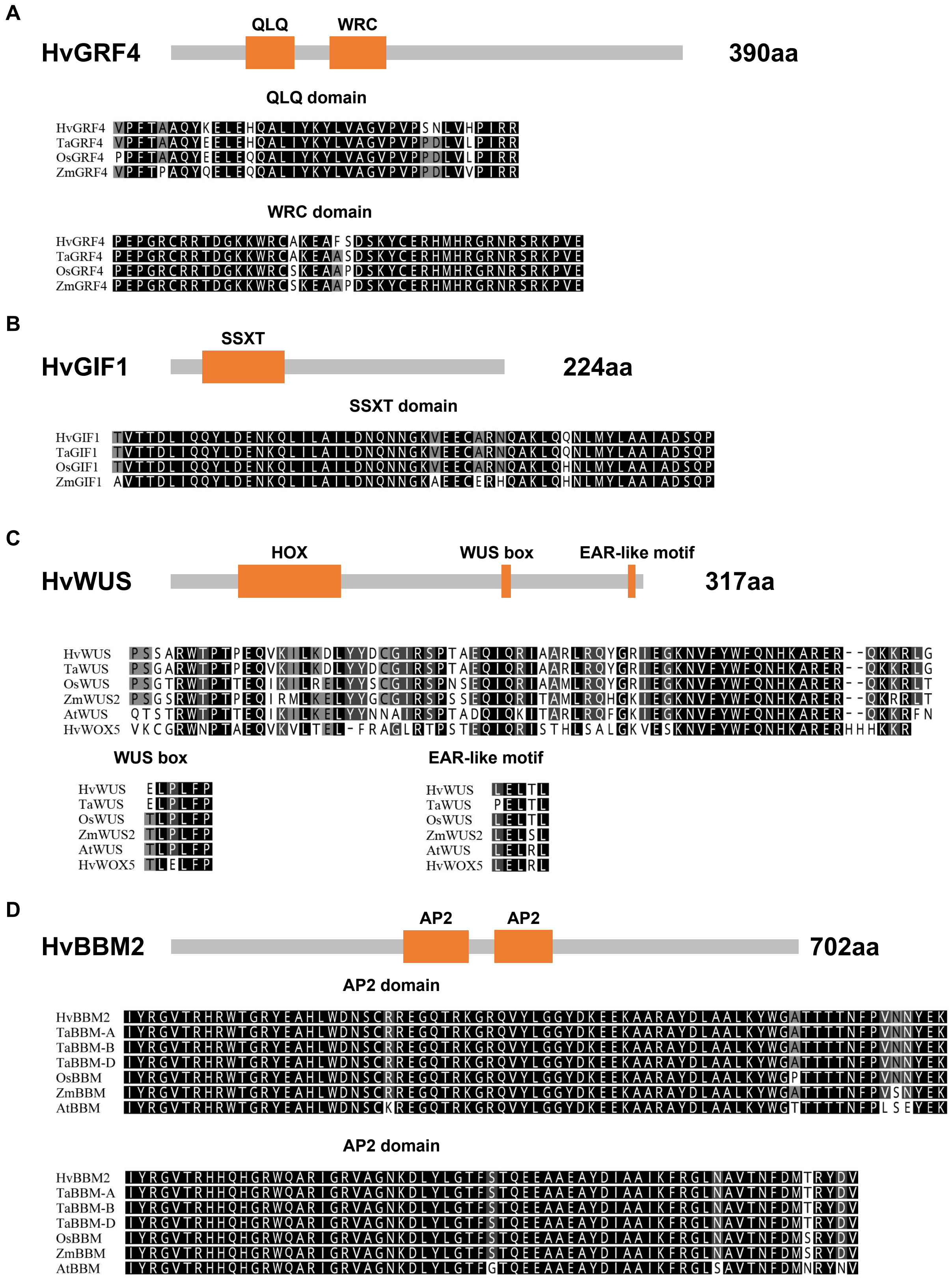

Supplementary Figure S2. Sequence alignment and domain analysis in barley, wheat, rice, maize, and Arabidopsis. **A)** GRF4. **B)** GIF1. **C)** WUS. **D)** BBM2.

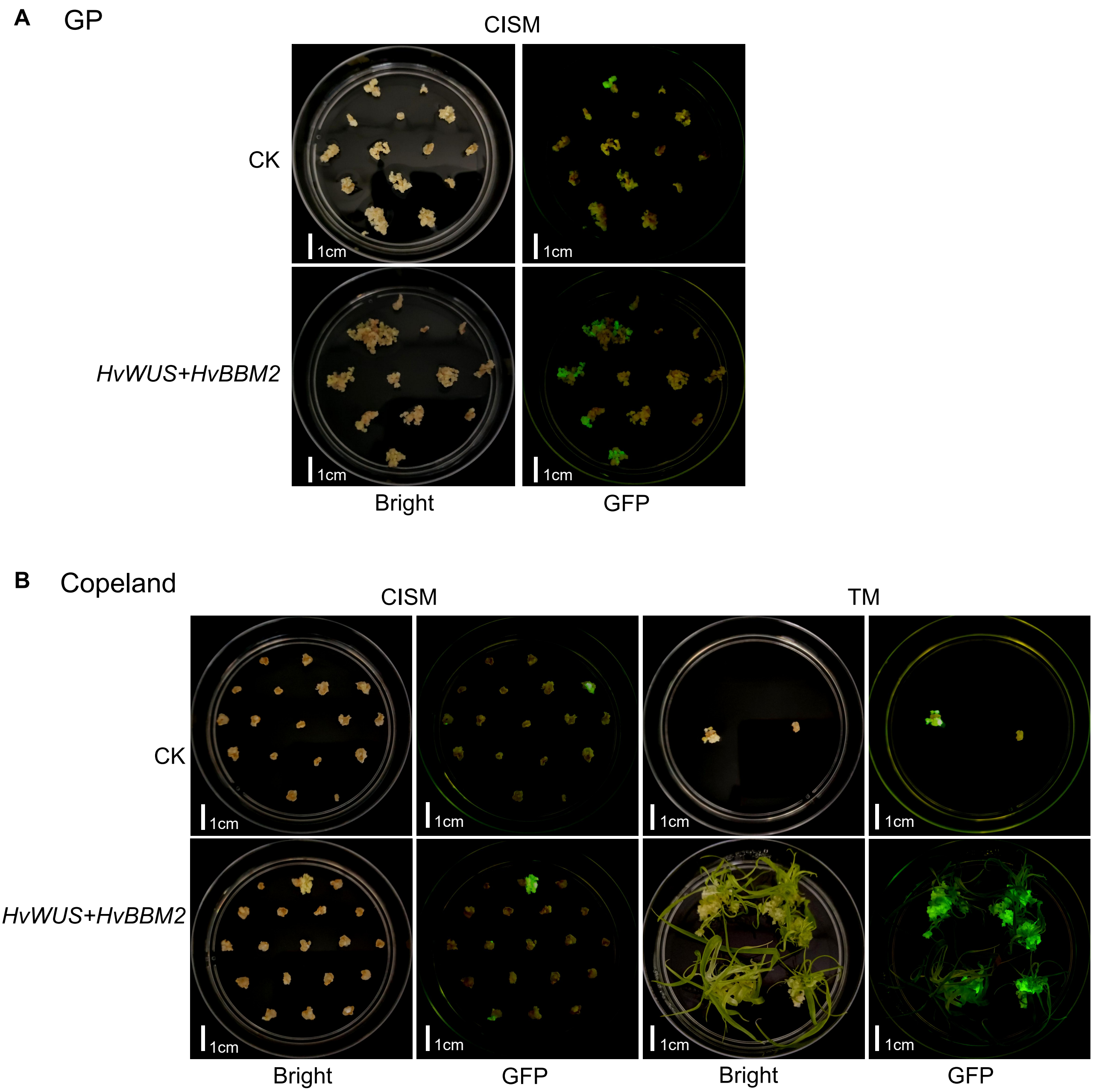

Supplementary Figure S3. Identification of positive callus through green fluorescence from ZsGreen. Fluorescence of positive callus transformed by control vector (CK) and *HvWUS+HvBBM2* in GP **A)** and Copeland **B)** on callus induction and selection medium (CISM), and transition medium (TM). The bright images for Copeland **B)** are the same as those shown in Figure 1E.

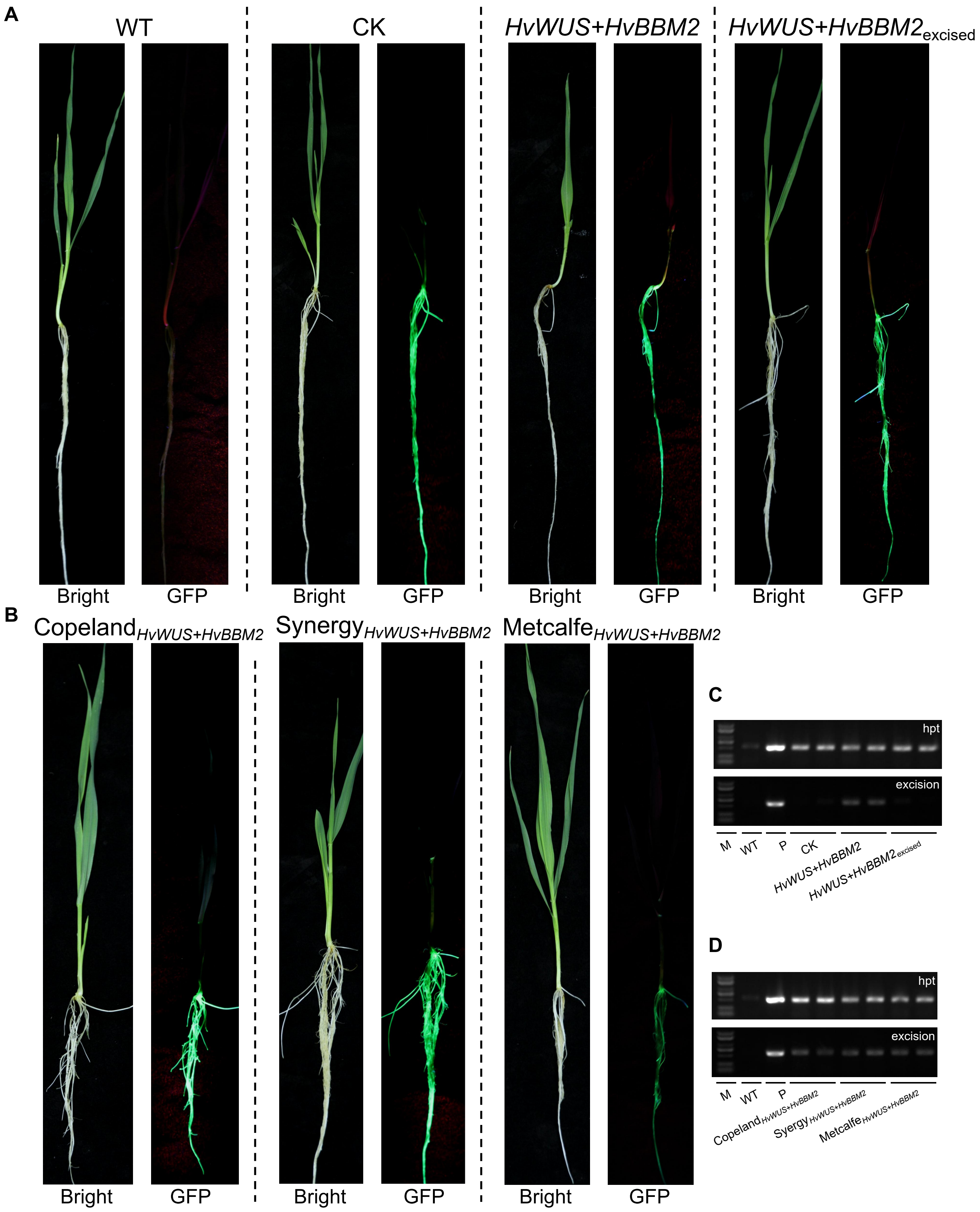

Supplementary Figure S4. Positive verification of transgenic T1 plants. **A)** Fluorescence of transgenic plants in GP. **B)** Fluorescence of transgenic plants in Copeland, Synergy and Metcalfe. **C)** PCR amplification for transgenic plants in GP. **D)** PCR amplification for transgenic plants in Copeland, Synergy and Metcalfe. P is the plasmid with pCAMBIA1300-*HvWUS*+*HvBBM2* vector.

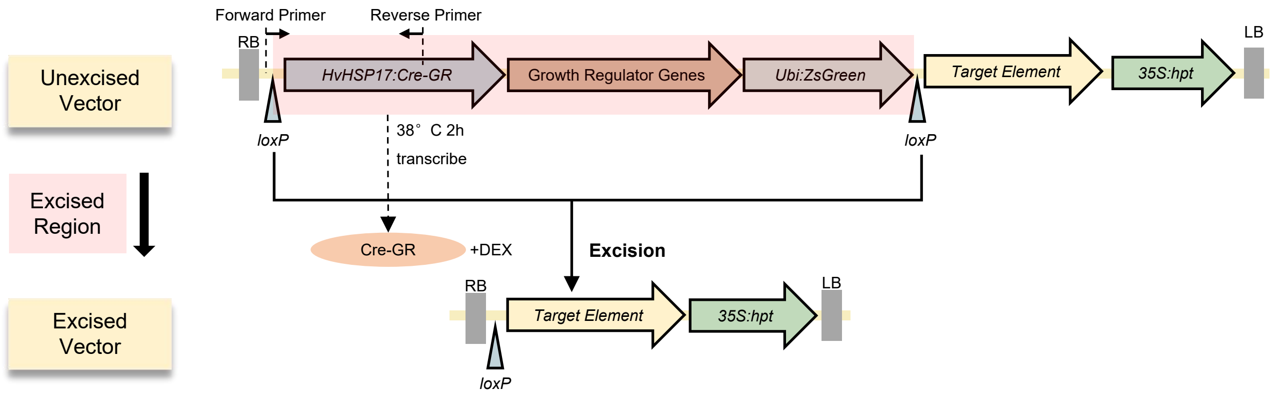

Supplementary Figure S5. Diagram of the Cre/*loxP* system.

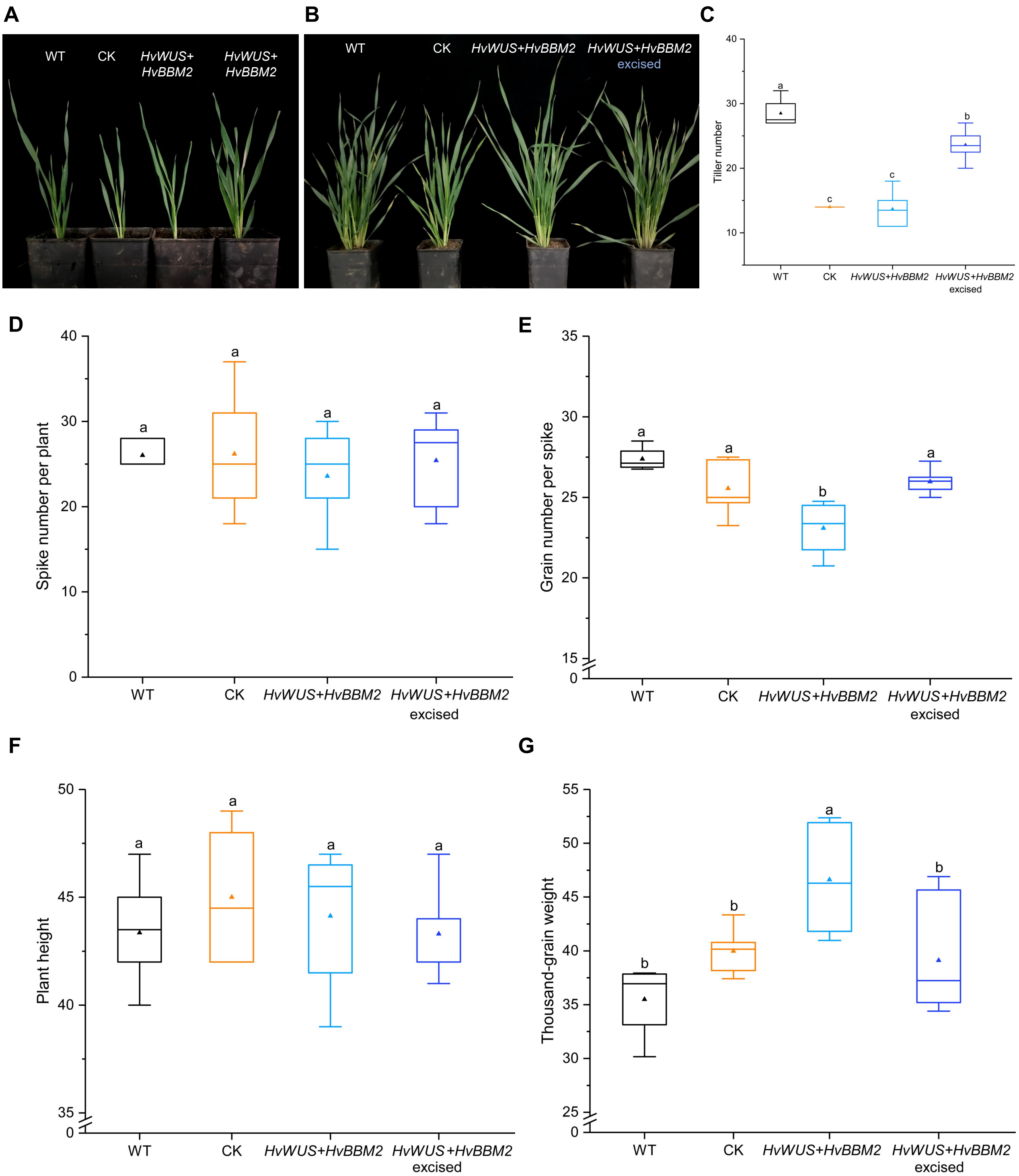

Supplementary Figure S6. Phenotype of wild-type GP as well as transgenic plants harboring CK, *HvWUS+HvBBM2*, or *HvWUS+HvBBM2*$_{\text{excised}}$. **A)** Seedling appearance. **B)** Tillering stage appearance. **C)** Tiller number (n = 2 to 8). **D)** Spike number per plant (n = 3 to 10). **E)** Grain number per spike (n = 4 to 7). **F)** Plant height (cm) (n = 6 to 14). **G)** Thousand-grain weight (g) (n = 4 to 7). Different letters indicate statistically significant differences at $p < 0.05$ based on one-way ANOVA. The box plots show median (center lines), interquartile range (box limits), and 1.5× interquartile range whisker extensions, while triangles indicate arithmetic means.

A

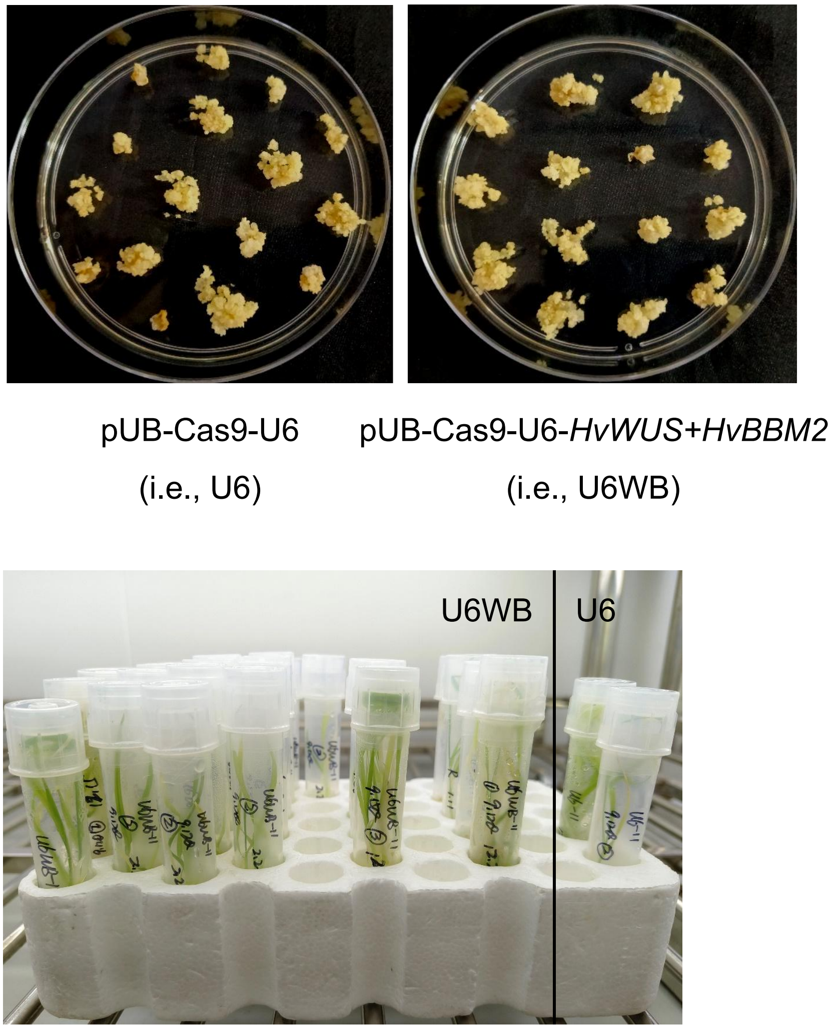

Supplementary Figure S7. Callus and regenerated plantlets transformed using pUB-Cas9-U6 (i.e., U6) or pUB-Cas9-U6-*HvWUS*+*HvBBM2* (i.e., U6WB). **A)** Callus. **B)** Regenerated plantlets.

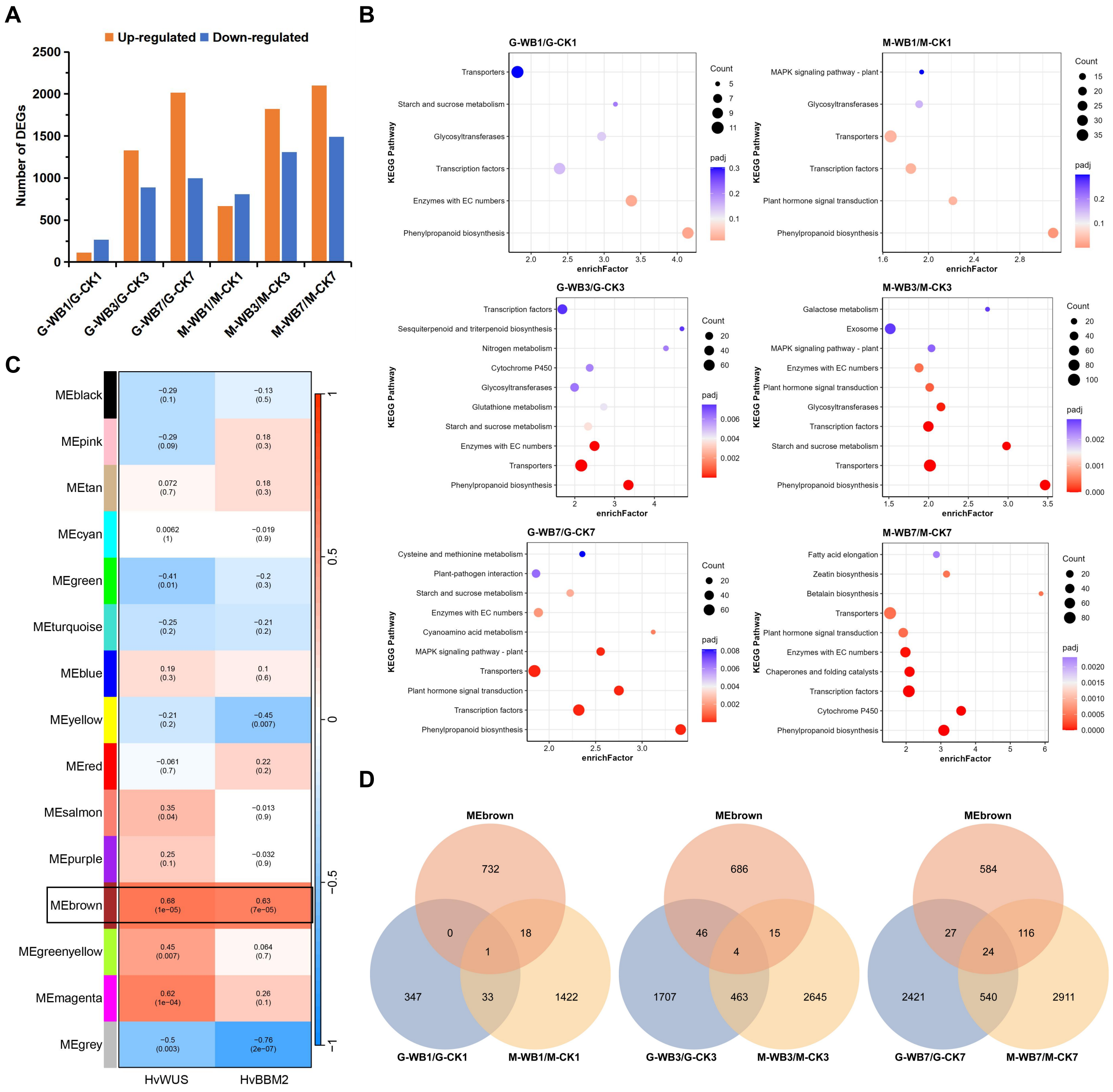

Supplementary Figure S8. Transcriptome analysis of callus transformed with CK or *HvWUS+HvBBM2* in the GP (G) or Metcalfe (M) background at three stages of culture. **A)** The number of differentially expressed gens (DEGs) between callus transformed with CK and *HvWUS+HvBBM2* (WB). **B)** Kyoto Encyclopedia of Genes and Genomes (KEGG) pathways of DEGs. **C)** The relationship between the modules identified by Weighted Gene Co-Expression Network Analysis (WGCNA) and the expression levels of *HvWUS* and *HvBBM2*. **D)** Venn diagrams of genes identified in MEbrown module of WGCNA and DEGs of WB/CK in GP and Metcalfe at 1 (left), 3 (middle), and 7 (right) weeks of culture.

Supplementary Table S1. Identification of plant growth regulators in barley.

Gene Name	Gene ID or GenBank ID
HvWUS	HORVU.MOREX.r3.2HG0201080
HvBBM2	HORVU.MOREX.r3.3HG0305370
HvWOX5	HORVU.MOREX.r3.3HG0301330
HvGRF4	HORVU.MOREX.r3.6HG0606810
HvGIF1	HORVU.MOREX.r3.4HG0339920
ZmWUS2	ABW43772.1
ZmBBM	CAJ29869.1
TaGRF4	TraesCS6A01G269600
TaGIF1	TraesCS4A01G250600

Supplementary Table S2. The detail information of ZU9, ZU10, Metcalfe, Synergy, and Copeland.

Variety	Full Name	Row Type	Country of Origin	Domestication Status	Growth Habit	Use
ZU9	Zhejing University NO.9	2	China	Cultivar	Semi-winter	Malting & Feeding
ZU10	Zhejing University NO.10	2	China	Cultivar	Semi-winter	Feeding
Metcalfe	AC Metcalfe	2	Canada	Cultivar	Spring	Malting
Synergy	AAC Synergy	2	Canada	Cultivar	Spring	Malting
Copeland	CDC Copeland	2	Canada	Cultivar	Spring	Malting

Supplementary Table S3. Summary of barley immature embryo transformation in ZU9, ZU10, Metcalfe, Synergy, and Copeland.

Variety	Experiment	Vector	No. of immature embryos	No. of induced calli	% of induced calli	No. of GFP positive calli	% of GFP positive calli	No. of positive plantlets	Freq. of positive plantlets	Transformation efficiency (%)
ZU9	1	CK	135	100	74.1	6	6.0	0	0.00	0.0
		HvWUS+HvBBM2	70	60	85.7	27	45.0	35	1.30	50.0
	2	CK	36	21	58.3	0	0.0	0	/	0
		HvWUS+HvBBM2	31	30	96.8	1	3.3	0	0.00	0.0
	3	CK	248	234	94.4	1	0.4	0	0.00	0.0
		HvWUS+HvBBM2	322	313	97.2	14	4.5	2	0.14	0.6
ZU10	1	CK	159	135	84.9	11	8.1	0	0.00	0.0
		HvWUS+HvBBM2	114	103	90.4	35	34.0	96	2.74	84.2
	2	CK	71	55	77.5	0	0.0	0	/	0
		HvWUS+HvBBM2	66	60	90.9	5	8.3	3	0.60	4.5
	3	CK	127	116	91.3	3	2.6	0	0.00	0.0
		HvWUS+HvBBM2	85	76	89.4	13	17.1	0	0.00	0.0
Metcalfe	1	CK	43	42	97.7	1	2.4	0	0.00	0.0
		HvWUS+HvBBM2	80	74	92.5	9	12.2	50	5.56	62.5
	2	CK	79	62	78.5	0	0.0	0	/	0
		HvWUS+HvBBM2	99	80	80.8	25	31.3	34	1.36	34.3

Synergy	1	CK	163	78	47.9	0	0.0	0	/	0
		HvWUS+HvBBM2	248	110	44.4	0	0.0	0	/	0
	2	CK	67	47	70.1	1	2.1	0	0.00	0.0
		HvWUS+HvBBM2	101	90	89.1	10	11.1	8	0.80	7.9
	3	CK	179	144	80.4	1	0.7	0	0.00	0.0
		HvWUS+HvBBM2	162	91	56.2	14	15.4	8	0.57	4.9
Copeland	1	CK	270	177	65.6	3	1.7	0	0.00	0.0
		HvWUS+HvBBM2	164	139	84.8	16	11.5	16	1.00	9.8
	2	CK	137	75	54.7	0	0.0	0	/	0
		HvWUS+HvBBM2	247	154	62.3	9	5.8	24	2.67	9.7
	3	CK	188	124	66.0	0	0.0	0	/	0
		HvWUS+HvBBM2	282	152	53.9	7	4.6	11	1.57	3.9

Supplementary Table S4. Detailed information on the candidate genes.

gene name	gene ID	annotation
MYB91	HORVU.MOREX.r3.5HG0441760	Myb transcription factor
MADS	HORVU.MOREX.r3.3HG0286170	MADS-box transcription factor
LAX2	HORVU.MOREX.r3.1HG0017310	Auxin influx transporter
WAT1	HORVU.MOREX.r3.3HG0219290	WAT1-related protein
ZPR3	HORVU.MOREX.r3.2HG0166000	Homeobox-leucine zipper protein HOX9 putative
AGL22	HORVU.MOREX.r3.4HG0406150	MADS box transcription factor
HSP70	HORVU.MOREX.r3.4HG0387610	70 kDa heat shock protein
CKI	HORVU.MOREX.r3.5HG0526800	cyclin-dependent kinase inhibitor
LEA6	HORVU.MOREX.r3.1HG0061780	Late embryogenesis abundant protein
AGL52	HORVU.MOREX.r3.5HG0428810	AGAMOUS-like 52
GASA4	HORVU.MOREX.r3.7HG0670790	Gibberellin-regulated family protein
NAC6	HORVU.MOREX.r3.2HG0111410	NAC domain protein
IAA3	HORVU.MOREX.r3.5HG0433440	Auxin-responsive protein
ARP/DRM	HORVU.MOREX.r3.4HG0376000	Auxin-repressed/dormancy-associated protein
ARP/DRM-2	HORVU.MOREX.r3.5HG0480830	Auxin repressed/dormancy associated protein
MYB94	HORVU.MOREX.r3.2HG0122400	Myb factor
IAA8	HORVU.MOREX.r3.1HG0086460	Auxin-responsive protein
ARF3	HORVU.MOREX.r3.3HG0289370	Auxin response factor
ARF3-2	HORVU.MOREX.r3.1HG0087670	Auxin response factor
